# BDNF Facilitates L-LTP Maintenance in the Absence of Protein Synthesis through PKMζ

**DOI:** 10.1371/journal.pone.0021568

**Published:** 2011-06-29

**Authors:** Fan Mei, Guhan Nagappan, Yang Ke, Todd C. Sacktor, Bai Lu

**Affiliations:** 1 School of Basic Medical Sciences, Peking University Health Science Center, Beijing, China; 2 Program in Developmental Neurobiology, *Eunice Kennedy Shriver*, National Institute of Child Health and Human Development, Bethesda, Maryland, United States of America; 3 GlaxoSmithKline, R&D China, Pudong, Shanghai, China; 4 The Robert F. Furchgott Center of Neural and Behavioural Science, Departments of Physiology and Pharmacology and Neurology, State University of New York (SUNY) Downstate Medical Center, Brooklyn, New York, United States of America; Medical College of Georgia, United States of America

## Abstract

Late-phase long term potentiation (L-LTP) is thought to be the cellular basis for long-term memory (LTM). While LTM as well as L-LTP is known to depend on transcription and translation, it is unclear why brain-derived neurotrophic factor (BDNF) could sustain L-LTP when protein synthesis is inhibited. The persistently active protein kinase ζ (PKMζ) is the only molecule implicated in perpetuating L-LTP maintenance. Here, in mouse acute brain slices, we show that inhibition of PKMζ reversed BDNF-dependent form of L-LTP. While BDNF did not alter the steady-state level of PKMζ, BDNF together with the L-LTP inducing theta-burst stimulation (TBS) increased PKMζ level even without protein synthesis. Finally, in the absence of *de novo* protein synthesis, BDNF maintained TBS-induced PKMζ at a sufficient level. These results suggest that BDNF sustains L-LTP through PKMζ in a protein synthesis-independent manner, revealing an unexpected link between BDNF and PKMζ.

## Introduction

LTP in acute hippocampus slices has long been used as a model to study the cellular mechanisms underlying learning and memory. There are temporally distinct types of LTP: protein synthesis-independent early phase LTP (E-LTP) and protein synthesis-dependent late phase LTP (L-LTP) [1], [2], [3], paralleling the two forms of memory – short-term and long-term memories [4]. While numerous studies have been done on E-LTP, much less is known about the mechanisms for L-LTP. The secreted trophic protein BDNF and intracellular signaling molecule PKMζ are the two best-studied molecules; both are necessary and sufficient to maintain L-LTP [5], [6], [7], [8], [9]. BDNF through its presynaptic or postsynaptic TrkB receptor activates the downstream mitogen-activated protein kinase (MAPK), phosphatidylinositol 3- kinase (PI3K) and phospholipase C-γ (PLC-γ) pathways [10]. PKMζ is a brain-specific, atypical isoform of protein kinase C. It is persistently active, due largely to the lack of regulatory domain and therefore second-messenger-independent [11]. BDNF and PKMζ share several common characteristics in regulating hippocampal L-LTP. First, either perfusion of BDNF or intracellular introduction of PKMζ directly facilitates synaptic transmission by promoting postsynaptic responses [9], [12], [13], [14]. Second, inhibition of either BDNF or PKMζ abolishes L-LTP [5], [15]. Third, BDNF and PKMζ could modulate the morphological changes of dendritic spines [12], [16]. However, the relationships between the two molecules in regulating L-LTP remain unclear.

Substantial evidence suggests that the expression of BDNF gene is controlled by neuronal activity [17]. In the hippocampus, the BDNF mRNA levels in the CA1 region are rapidly increased in response to the L-LTP inducing tetanic stimulation [18], [19]. Weak tetanic stimulation, which normally induces only E-LTP, could induce L-LTP as long as the BDNF levels are elevated [5]. With these results, one can hypothesize that strong tetani trigger the expression of BDNF which in turn enhances the synthesis of PKMζ in the hippocampus, leading to L-LTP. However, application of BDNF could rescue L-LTP deficits even when protein synthesis is completely blocked [5]. These perplexing results raise the possibility that BDNF may increase the PKMζ level not by enhancing its synthesis but by reducing degradation to achieve LTP maintenance.

The present study attempts to reveal a mechanistic link between BDNF and PKMζ. We found that BDNF-related neuronal activities augmented PKMζ expression but BDNF alone did not modulate steady-state PKMζ protein level. Moreover, in the absence of protein synthesis, BDNF sustained L-LTP by maintaining activity-induced PKMζ at a sufficient level. These results together suggest that BDNF-dependent L-LTP is mediated by PKMζ, and explain how BDNF can maintain L-LTP even when protein synthesis is completely blocked.

## Results

### PKMζ mediates BDNF-dependent late phase LTP

**Figure 1:**
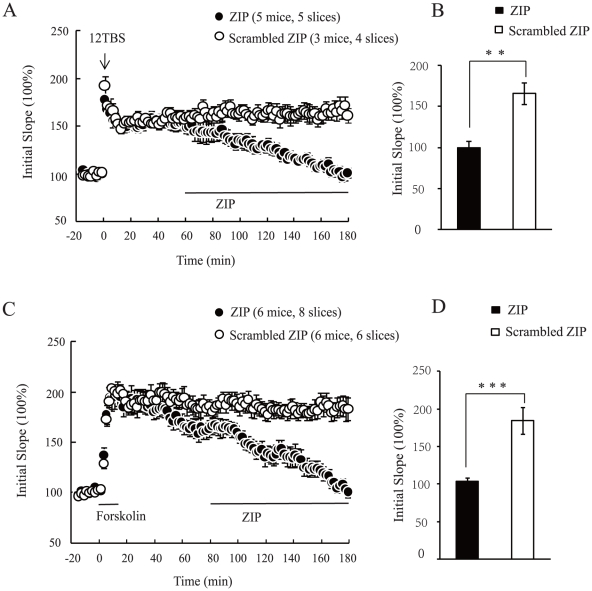
BDNF-dependent late phase LTP is mediated by PKMζ. (A, B) 12 TBS-induced L-LTP was reversed by PKMζ inhibitor ZIP. Field EPSP (fEPSP) was evoked in CA1 stratum radiatum by stimulating Schaffer Collateral in adult C57BL/6 mice. (A) After a stable baseline was obtained, 12 TBS was conducted. LTP was sustained at least for 3 hours. ZIP (5 µM) or scrambled ZIP peptide (5 µM) was applied at 1 hour after stimulation. (B) Quantification of the initial slope value from the last 5 minutes recording. (C, D) Forskolin-induced L-LTP was abolished by PKMζ inhibitor ZIP. The experiments were done identically as in (A), except that L-LTP was induced by a transient perfusion of forskolin (50 µM) and IBMX (30 µM) for 15 minutes. ZIP or scrambled ZIP was applied at 80 minutes after chemical induction when stable L-LTP was fully established. Numbers of slices and mice used in each condition are indicated at the top of each plot. In this and all other figures, data are presented as mean ± s.e.m. * p<0.05, ** p<0.01,*** p<0.001, Student's t-Test.

Previous studies indicate that L-LTP can be further divided into the BDNF-dependent form which can be induced by theta burst stimulation (12 TBS) or a perfusion of cAMP analogs such as forskolin, and the BDNF-independent form which is triggered by the classic 4 sets of tetanus (4×tetani) [20]. PKMζ is known to mediate L-LTP induced by 4 tetani [21]. To determine whether PKMζ is also responsible for BDNF-dependent form of L-LTP, we applied ZIP, a myristoylated PKMζ-substrate peptide inhibitor (5 µM), well after the BDNF-dependent L-LTP was expressed. In 12 TBS-induced L-LTP, ZIP applied 1 hour after stimulation successfully reversed L-LTP (Fig. 1A, 1B, 99±8% at 175–180 min, p<0.01 compared with control). In contrast, a scrambled ZIP peptide (5 µM) did not affect L-LTP maintenance (166±13% at 175–180 min). Forskolin-induced L-LTP was induced by a 15-min perfusion of a combined forskolin (50 µM) and the phosphodiesterase inhibitor IBMX (30 µM). ZIP was applied 80 min after LTP induction when a stable L-LTP was fully established. Again, ZIP abolished L-LTP (Fig. 1C, 1D, 103±6% for ZIP, 184±18% for Scrambled ZIP, at 175–180 min, p<0.001). These results suggest that PKMζ is required for the maintenance of BDNF-dependent L-LTP.

### Steady-State PKMζ level is not maintained by BDNF

**Figure 2:**
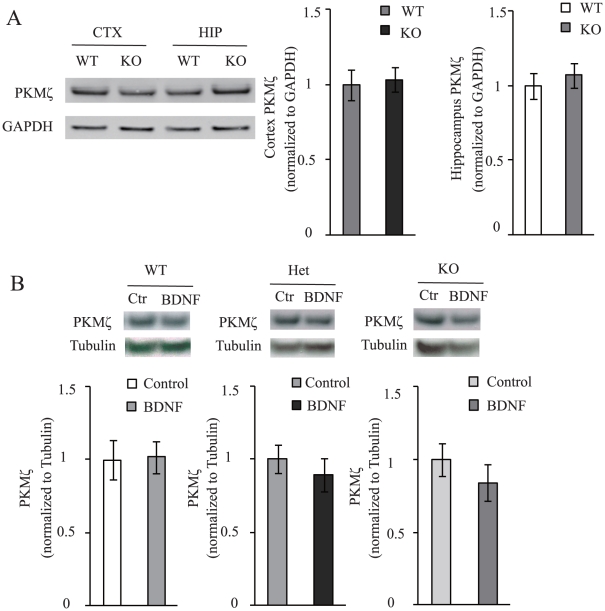
Steady-State PKMζ protein level is not regulated by BDNF. (A) PKMζ protein level in cortex and hippocampus of BDNF KO and WT littermates. At postnatal day 18, cortex or hippocampus from BDNF KO and WT littermates were dissected and subjected to Western blot. Representative blots and quantification of data were shown. GAPDH was used as loading control. (n = 5–8 independent experiments). (B) PKMζ expression in primary neuron cultures derived from different genotypes after BDNF treatment. DIV 7 cortical primary cultures of WT, Het or KO genotype were separately treated with BDNF (100 ng/ml) or vehicle for 24 hours. For each experiment, BDNF treatment group was normalized against vehicle treatment group. Representative blots are shown on top of the quantification of data. (n = 5 independent experiments).

To investigate whether and how BDNF regulates PKMζ expression, we tested steady-state PKMζ expression in wild-type (WT) and homozygous BDNF knockout (KO) mice. At postnatal day 18 (P18), brain tissues were dissected and subjected to Western blot analysis. Surprisingly, there was no significant difference of endogenous PKMζ expression between WT and KO mice in the cortex and hippocampus, respectively (Fig. 2A, Cortex, p = 0.79; Hippocampus, p = 0.52).

A number of studies have demonstrated that BDNF promotes gene transcription and translation [22]. We next investigated whether exogenous BDNF treatment affected PKMζ protein level in primary neuron culture. Embryonic neurons derived from WT, heterozygous (Het) and KO mice were cultured for 7 days (DIV 7) and then exposed to BDNF (100 ng/ml) or vehicle for 24 hours, and total PKMζ protein level was measured by Western blot. Addition of BDNF did not cause any significant change in the levels of PKMζ in WT or BDNF mutant genotypes (Fig. 2B, WT, p = 0.91; Het, p = 0.44; KO, p = 0.34). Thus, it appears that without substantial enhancement of neuronal or synaptic activities, BDNF does not alter the steady-state level of PKMζ protein.

### BDNF modulates activity-dependent PKMζ levels to sustain L-LTP in the absence of protein synthesis

**Figure 3:**
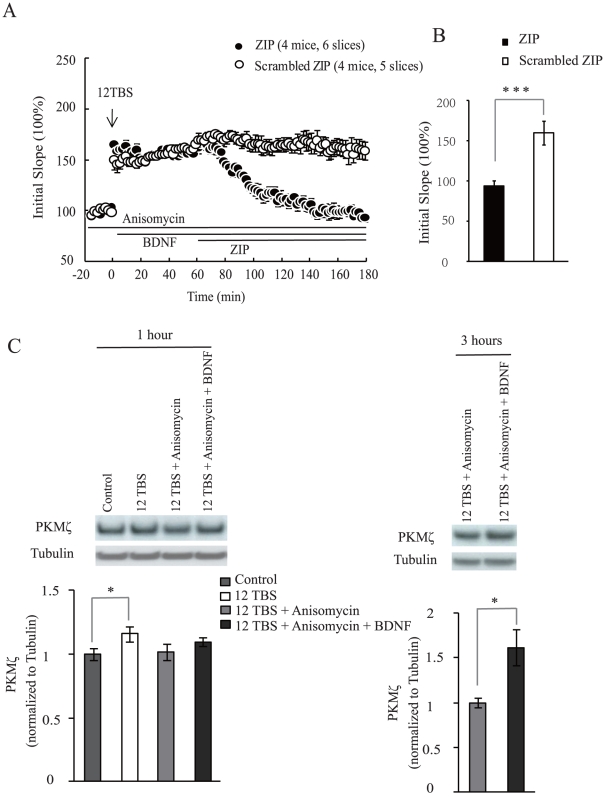
BDNF modulates activity-dependent PKMζ level to sustain L-LTP in the absence of protein synthesis. (A, B) Rescuing L-LTP impairment by BDNF in the presence of anisomycin is PKMζ-dependent. (A) Applications of various drugs were indicated by horizontal bars. Anisomycin (40 µM) was used throughout the entire experiment. BDNF (200 ng/ml) was applied 3 minutes after 12TBS stimulation and successfully rescued L-LTP impairment. ZIP was applied at 1 hour after stimulation and completely abolished L-LTP. (B) Quantification of the initial slope from the last 5 minutes of recording. (C) PKMζ protein level of hippocampal CA1 derived from WT mice at 1 hour and 3 hours after 12TBS stimulation. Tubulin was used as loading control. The 12 TBS group was normalized against control group. The 12 TBS plus BDNF and anisomycin treatment groups were normalized against that without BDNF treatment. Representative blots are shown on top of the quantification of data (3–5 slices per treatment, n = 3 independent experiments).

We have previously shown that treatment with BDNF is sufficient to rescue L-LTP impairment when protein synthesis is completely blocked [5]. We attempted to examine whether BDNF could modulate PKMζ to sustain the L-LTP at this situation. Consistent with the previous study, L-LTP was fully established by BDNF (200 ng/ml) despite of protein synthesis inhibition by anisomycin (40 µM). We next applied PKMζ inhibitor ZIP at 1 hour after tetanus and found L-LTP was completely reversed (Fig. 3A–3B, 94±6% for ZIP, 160±15% for Scrambled ZIP, at 175–180 min, p<0.001). These results raise the possibility that BDNF regulates PKMζ to ensure a sustained L-LTP through a protein synthesis independent mechanism.

To further characterize how BDNF regulates PKMζ, we compared PKMζ level in the hippocampal slices at different time points after 12 TBS. The 12 TBS group was normalized against control condition in which slices were not stimulated. The 12 TBS plus BDNF and anisomycin treatment groups were normalized against that without BDNF treatment. The PKMζ signals on the Western blot were normalized to that of β-tubulin on the same lane. At the early stage of L-LTP (around 1 hour after tetanus), synaptic activation induced a small but statistically significant increase of PKMζ level (Fig. 3C, 116±5.8%, p<0.05). This elevation of PKMζ level was protein synthesis dependent. Application of BDNF (200 ng/ml) together with 12TBS did not further increase PKMζ level. At the late stage of L-LTP (around 3 hour after tetanus), the BDNF-treated slices exhibited a much higher level of PKMζ compared with the one in the presence of anisomycin (Fig. 3C, 161.6±20.5%, p<0.05). Thus, BDNF combined with strong tetanus could increase the steady-state level of PKMζ when protein synthesis is completely blocked. These results suggest that in the absence of protein synthesis strong tetanic stimulation together with BDNF could somehow elevate PKMζ protein level, which in turn is responsible for L-LTP maintenance.

## Discussion

Although L-LTP is known to be dependent on translation, it has been puzzling why BDNF could rescue the L- LTP deficit in the presence of the protein synthesis inhibitor anisomycin [5]. Considering that anisomycin may elicit unspecific stress-related pathways [23], we previously also applied emetine (20 µM) to block protein synthesis. A similar rescuing effect of BDNF was observed in L-LTP impairment, validating the notion that BDNF promotes L-LTP maintenance in the absence of protein synthesis [5]. In the present study, we have revealed an unexpected role of PKMζ in mediating this BDNF-dependent form of L-LTP. In the absence of protein synthesis, BDNF seems to sustain L-LTP by means of maintaining a sufficient level of activity-induced PKMζ. These data provide a mechanistic link between BDNF and PKMζ and suggest their critical role in the maintenance of L-LTP despite of protein synthesis inhibition.

Unlike the classic, tetanus-induced L-LTP, the cAMP or 12TBS induced L-LTP requires an increase in local concentration of dendritic proteins but not nucleus activity [24], and is dependent on BDNF [20]. Similar to the classic L-LTP, however, we now demonstrate that the BDNF-dependent L-LTP also requires PKMζ. Interestingly, in primed L-LTP through type I mGluRs activation, neither suppression of BDNF nor PKMζ alone could reverse L-LTP. But a co-inhibition of BDNF and PKMζ completely abolishes its maintenance [25]. These results could be interpreted as PKMζ acts either in parallel or synergistically with BDNF. However, we provide several lines of evidence suggesting that PKMζ could be downstream of BDNF, at least in BDNF-dependent L-LTP. First, application of the PKMζ inhibitor ZIP after cAMP or 12 TBS reverses the BDNF-dependent LTP. Second, BDNF together with 12TBS increases hippocampal PKMζ level. Finally, in the presence of anisomycin, BDNF rescue of L-LTP deficit could be reversed by ZIP.

In general, BDNF-TrkB signaling is crucial for activity-induced new protein synthesis [22]. Moreover, synthesis of PKMζ is a common target of many signaling pathways in LTP induction, including the major BDNF downstream pathways, such as PI3-kinase, MAPK, mTOR, etc [26], [27]. However, we did not detect a difference of steady-state PKMζ expression between WT and BDNF KO mice. Further, application of BDNF to cultured WT neurons did not increase PKMζ protein level. We reasoned that BDNF may need to work together with high frequency neuronal activity to up-regulate PKMζ through a protein synthesis independent mechanism. Indeed, we found that BDNF together with the L-LTP inducing 12TBS increases the PKMζ protein level, even in the presence of anisomycin.

How BDNF maintains PKMζ level without protein synthesis? One attractive hypothesis is that BDNF inhibits TBS-induced degradation of PKMζ through the ubiquitin-proteasome system (UPS). A balance in protein synthesis and degradation has been implicated in the maintenance of long term plasticity, structurally and functionally [28]. When protein synthesis is inhibited, PKMζ level decreases primarily through UPS-mediated degradation. Given that BDNF-TrkB signaling acts upstream of UPS coupling neuronal activity with protein turnover [29], it is possible that BDNF counters PKMζ degradation to maintain L-LTP. Indeed, without BDNF treatment, PKMζ level keeps low under anisomycin perfusion [30]. Moreover, there is a critical window for BDNF to rescue L-LTP impairment — no later than 10 minutes after tetanus [5]. An alternative hypothesis is that BDNF regulates PKMζ protein translocation to the stimulated synaptic site. According to “synaptic tagging” theory, PKMζ is suggested as a plasticity-related protein (PRP) that not only potentiate synaptic responses at strongly tetanized pathways, but also at weakly stimulated pathways as long as synaptic tags are set [31]. BDNF may facilitate PKMζ translocation from cytoplasm to synaptic sites. Specifically, when protein synthesis is inhibited, a local shortage of newly synthesized PKMζ may drive the need for PKMζ translocation and BDNF may facilitate this process. Regardless, it is critical for BDNF to hold PKMζ at a sufficient level within this window before it is completely consumed by dynamic neuronal activities.

Taken together, these results expand the range of BDNF modulation of long term plasticity beyond a protein synthesis dependent manner and provide a strong mechanistic link between BDNF and PKMζ in the maintenance of L- LTP.

## Materials and Methods

### Ethics Statement

All experiments were approved by the National Institutes of Health (NIH) Animal Care and Use Committee and followed the NIH Guidelines “Using Animals in Intramural Research”. The NICHD Animal Use Proposal Number is 07-020.

### Animals

Homozygous BDNF knockout mice (KO), and wild type (WT) littermates were derived from BDNF heterozygous breeding pairs in C57BL/6 background as described [32].

### Western Blotting

Brain tissues or primary neuron cultures were lysed in RIPA buffer containing (in mM): 50 Tris-HCl (pH 7.4), 150 NaCl, 2 EDTA, 1% IGEPAL, 0.1% SDS, a cocktail of protease inhibitor (Calbiochem, San Diego, CA) and phosphatase inhibitor (Calbiochem, San Diego, CA). Lysates were homogenized by sonication. Supernatants were collected after centrifugation at 13,200 rpm for 15 min at 4°C. Protein concentration was measured by Bio-Rad DC protein assay (Bio-Rad, Hercules, CA). For Western blot analysis, protein samples were mixed with LDS sample buffer (Invitrogen, Carlsbad, CA) and separated by Bis-Tris 4–12% gel (Invitrogen, Carlsbad, CA). Proteins were transferred to a PVDF membrane by iBlot (Invitrogen, Carlsbad, CA). After blocking in Tris buffer saline-1% non-fat dry milk for 1 hour, membranes were probed with rabbit anti-PKMζ (C-terminal, 1∶500, kindly provided by Dr. Sacktor Todd) overnight at 4°C. HRP-conjugated secondary antibody (Pierce, Rockford, IL) was used for detection in a chemiluminescent system. Glyceraldehydes-3-phosphate dehydrogenase (GAPDH) (1∶10,000, Abcam, Cambridge, MA) or Tubulin (1∶5000, Abcam, Cambridge, MA) was used as loading control in the same sample. Densitometric analysis was conducted using ImageJ software (NIH, Bethesda, MD). All experiments were repeated at least 3 times (*n* = 3), using independent samples.

### Primary Neuron Culture

Primary cortical neurons were cultured from embryos produced by crossing BDNF heterozygous animals. Each fetus (E18) was dissected carefully to prevent blood contamination. A tissue chunk was pinched off for genotyping. Cortices from fetuses of the same genotype were digested with trypsin, dissociated and plated together. At DIV 7, vehicle or BDNF (100 ng/ml) was applied to cultures for 24 hours.

### Electrophysiological Recording

Animals (6–10-week old, in C57BL/6 background) were anesthetized and decapitated. Brains were placed in ice-cold high Mg^2+^ artificial cerebrospinal fluid (ACSF) (in mM: 124 NaCl, 26.2 NaHCO_3_, 1 NaH_2_PO_4_, 4.4 KCl, 1.25 CaCl_2_, 2.6 MgSO_4_, 10 D-Glucose) bubbled with 95% O_2_ and 5% CO_2_. Transverse hippocampus slices (400 µm thick) were prepared with a vibrating microtome (Leica, Germany). The slices were stored submerged in recording ACSF (124 NaCl, 26.2 NaHCO_3_, 1 NaH_2_PO_4_, 4.4 KCl, 2.5 CaCl_2_, 1.3 MgSO_4_, 10 D-Glucose) for 30 minutes at 34°C and 30 minutes at room temperature. Recording was made in a submersion chamber (30°C, flow rate around 2 ml/min) perfused with recording ACSF.

Field excitatory postsynaptic potentials (fEPSP) were evoked in CA1 stratum radiatum by stimulating Schaffer Collateral with a bipolar tungsten electrode and recorded with ACSF-filled glass pipettes using an Axoclamp-2B amplifier (Axon Instruments, Sunnyvale, CA). Recordings with maximal fEPSP less than 1 mV or with substantial changes in the fiber volley were rejected. Baseline responses were set to ∼40% of maximal response and were recorded for 15 minutes. Late phase long-term potentiation was induced by tetanic stimulation, which contains 12 bursts, each with 4 pulses at 100 Hz and an inter-burst interval of 200 msec.

For pharmacologically induced long-term potentiation, forskolin (50 µM, Sigma, St. Louis, MO) and IBMX (30 µM, Sigma, St. Louis, MO) were applied in bath for 15 minutes and washed out with recording ACSF. The myristoylated zeta-pseudosubstrate peptide (ZIP, 5 µM, myr-SIYRRGARRWRKL-OH, Invitrogen, Carlsbad, CA) and its corresponding scrambled control peptide (5 µM, myr-RLYRKRIWRSAGR-OH, Invitrogen, Carlsbad, CA) were dissolved in Dimethylsulfoxide (DMSO). ZIP and scrambled ZIP were applied to the bath 1 hour after stimulation.

The initial slope of the fEPSP was measured as an index of synaptic strength. Data was analyzed by Clampfit 9 (Molecular Devices, Sunnyvale, CA) and presented as mean ± s.e.m..
